# Parameter Tuning Patterns for Random Graph Coloring with Quantum Annealing

**DOI:** 10.1371/journal.pone.0050060

**Published:** 2012-11-14

**Authors:** Olawale Titiloye, Alan Crispin

**Affiliations:** School of Computing, Mathematics and Digital Technology, Manchester Metropolitan University, Manchester, United Kingdom; University of Nottingham, United Kingdom

## Abstract

Quantum annealing is a combinatorial optimization technique inspired by quantum mechanics. Here we show that a spin model for the *k*-coloring of large dense random graphs can be field tuned so that its acceptance ratio diverges during Monte Carlo quantum annealing, until a ground state is reached. We also find that simulations exhibiting such a diverging acceptance ratio are generally more effective than those tuned to the more conventional pattern of a declining and/or stagnating acceptance ratio. This observation facilitates the discovery of solutions to several well-known benchmark *k*-coloring instances, some of which have been open for almost two decades.

## Introduction

Quantum annealing [1]–[6] is a combinatorial optimization technique that employs a quantum fluctuation parameter 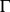 for the purpose of escaping local minima. The parameter 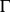 is often a transverse magnetic field in the presence of a low temperature 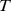 [3]. Quantum annealing studies have been carried out on NP-hard [7] problems such as the traveling salesman problem [8] and the graph coloring problem [9], [10]. Our version of quantum annealing is close to that in ref. [8] in terms of the approximations employed in the formulation. The algorithms in refs. [2], [5] prioritize simulating a quantum system as strictly as possible, while ours is more flexible in incorporating known classical optimization techniques, with the main aim of solving large and difficult combinatorial optimization problems on a classical computer. The graph *k*-coloring problem requires a determination of whether every vertex of a given graph can be colored differently to all its adjacent vertices, when only *k* colors are available. In order to get the best performance out of quantum annealing, the main parameters 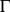 and 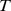 need to be tuned according to the particular problem instance under consideration. Although suitable values for the parameters can usually be found by trial runs [8], [9], a study of why certain values work better than others is desirable. Our findings show that the best parameter settings for dense random graphs are those that induce a continuously increasing acceptance ratio during Monte Carlo quantum annealing. Achieving this involves setting a low value for 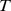, and tuning 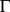 to within a critical range.

## Methods

Given an undirected graph 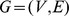 with a vertex set 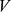
of size 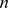, an edge set 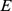, and a set 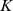 consisting of 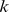 colors, 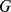 is said to be *k*-colorable if there exists a mapping 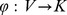 such that 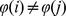, for all 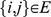. To decide *k*-colorability, we can minimize a cost function or problem Hamiltonian 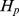, given by the number of edges with conflicts. A graph is *k*-colorable if and only if some configuration 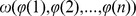 exists such that 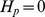. Any 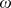 with 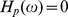 is also a global minimum. A configuration 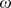 can be expressed in terms of 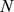
constrained Boolean variables 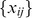, where 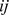 represents a pair of vertices with 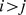, and 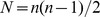 [9]. The Boolean variables are such that 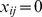 when 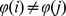, and 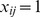 otherwise [9], [10]. To create an Ising model, we define the set of spins 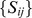, where 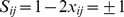. When the spins are defined this way, not all of the 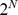
possible spin configurations correspond to a valid member of the *k*-coloring search space. Therefore the *k*-coloring search space cannot be explored by starting from just any spin configuration, and moving to a different one with a single spin flip. Instead, we first obtain an initial 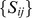 from a uniformly random assignment of one of the *k* colors to each of the vertices. Afterwards, a new valid configuration can be obtained by changing the color of a vertex, thereby implicitly performing specific multiple spin flips that maintain validity. The problem Hamiltonian can be represented as

**Figure 1:**
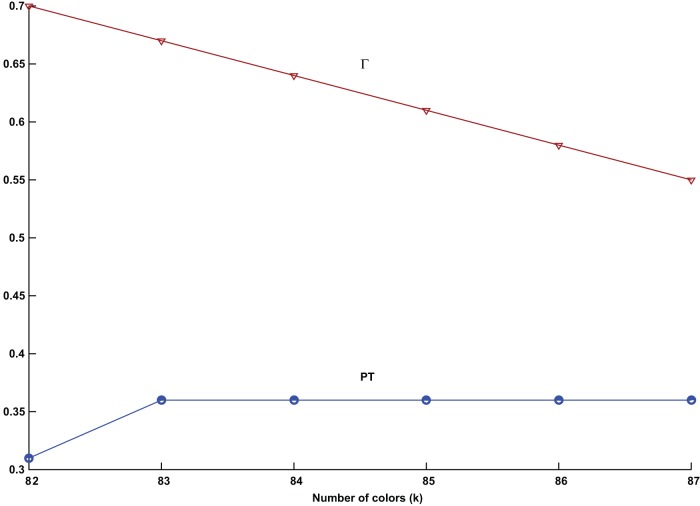
Parameter tuning variance with the number of colors for the graph DSJC1000.5. The field strength *Γ* (red), and the effective temperature *PT* (blue), plotted against the number of available colors *k*.



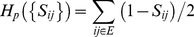
(1)The classical Hamiltonian in equation (1) can be converted to a quantum Hamiltonian with a transverse field to give
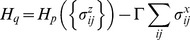
(2)where 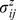 and 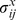 are Pauli matrices, and 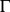 is a parameter representing the strength of a magnetic field providing quantum fluctuations. To address large problem instances feasibly on a classical computer, we use path integral Monte Carlo. This involves applying a Suzuki-Trotter transformation to 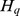 in equation (2) to give a new classical Hamiltonian.



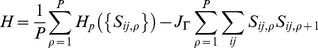
(3)The Hamiltonian 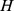 in equation (3) consists of 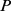 replicas of the original problem Hamiltonian, simulated at a fixed effective temperature 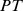, with 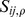 representing the 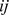-th spin of the 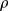-th replica, and 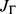 given as 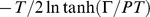 [9], [11]. Just as in the quantum annealing of the traveling salesman problem [8], the use of a single spin-flip transverse term in equation (2) is an approximation enabling a straightforward application of the Suzuki-Trotter transformation. In our implementation, only conflicting vertices are eligible for a color change with the Metropolis criterion [9], [10].

**Figure 2:**
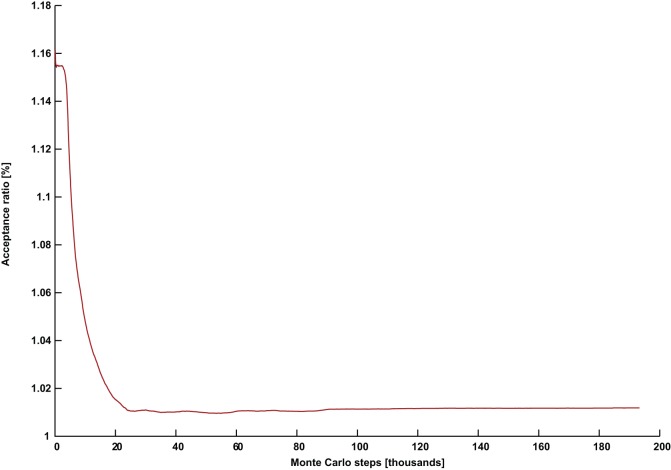
An unsuccessful simulation with a declining and stagnating acceptance ratio. The acceptance ratio plotted against Monte Carlo steps for (*G* = DSJC1000.5, *k* = 82) with parameters *Γ* = 0.7 and *PT* = 0.36.

**Figure 3:**
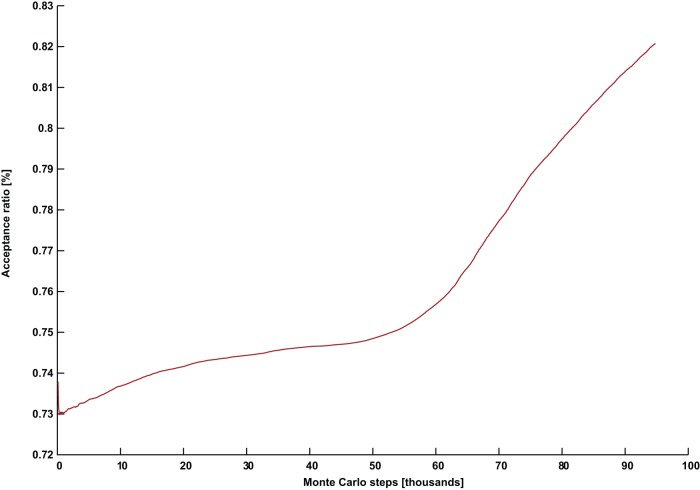
A successful simulation with a continuously rising acceptance ratio. The acceptance ratio plotted against Monte Carlo steps for (*G* = DSJC1000.5, *k* = 82) with parameters *Γ* = 0.7 and *PT* = 0.31.

Parameter tuning is crucial to the success of quantum annealing. In addition to the tuning of the temperature 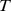 and the field strength 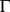, a decrement rate for 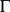 is usually required [8], [9], [11], [12]. If the initial value of 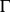 is too high, then a slow decrement rate can become impractical. On the other hand, decreasing 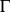 too quickly can result in the system being trapped by local minima. But if 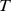 and 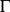 are heuristically chosen to fit the problem instance, we have recently experimentally demonstrated for graph coloring that successful simulations can be achieved with 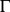 fixed throughout the duration of a particular simulation [10]. This is the variant of quantum annealing that we use in the current work. A good value of 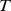 for a problem instance 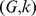 is usually a suitable value for the more difficult instance 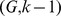. Additionally, a good value of 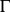 for 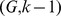 can usually be obtained from that of 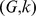 by incrementing it slightly [9], [10].

**Table 1:** Quantum annealing (QA) coloring results compared with the best algorithms.

Graph	QA	Evolutionary-Tabu	Extraction pre- processing
DSJC500.5	**47**	48 refs. [17], [21], [23], [24]	48 ref. [22]
DSJC1000.5	**82**	83 refs. [17], [21], [23], [24]	83 ref. [26]
DSJC1000.9	**222**	223 refs. [21], [24]	222 ref. [26]
C2000.5	**145**	148 refs. [21], [24]	145 ref. [27]
C4000.5	262 (**259** *)	271 ref. [21]	259 ref. [27]
C2000.9	**400**	413 ref. [26]	408 ref. [27]
flat1000_76_0	**81**	82 refs. [21], [23], [24]	81 ref. [27]

* We found 259-colorings for C4000.5 by performing quantum annealing on a residual graph obtained from the independent set extraction experiments in ref. [26].

**Table 2:** Detailed quantum annealing results.

Graph	k	PT	Γ	Attempted	Accepted	Time	Success
DSJC500.5	**47**	0.30	0.70	2.8×10^11^	4.1×10^9^	36 min	2/10
DSJC1000.5	**82**	0.31	0.70	5.6×10^11^	4.4×10^9^	1.2 hr	10/10
DSJC1000.9	**222**	0.20	0.40	5.9×10^11^	3.1×10^9^	1.1 hr	6/10
C2000.5	146	0.32	0.65	2.5×10^12^	1.1×10^10^	5.4 hr	5/5
	**145**	0.32	0.69	1.4×10^13^	6.7×10^10^	31.6 hr	2/2
C4000.5	270	0.28	0.51	1.2×10^14^	2.0×10^11^	11 days	1/1
	262	0.28	0.57	1.3×10^15^	1.8×10^12^	4 mo.	1/1
C2000.9	403	0.18	0.29	1.2×10^13^	2.0×10^10^	24 hr	5/5
	402	0.18	0.295	2.8×10^13^	4.5×10^10^	54 hr	1/1
	401	0.18	0.30	8.8×10^13^	1.4×10^11^	174 hr	1/1
	**400**	0.18	0.31	1.3×10^14^	2.0×10^11^	505 hr	1/1
flat1000_76_0	**81**	0.31	0.70	1.0×10^12^	8.7×10^9^	2.2 hr	10/10

Columns 5 and 6 contain the average number of attempted and accepted color changes respectively for successful runs. The average time for a successful simulation is displayed in column 7, while the success rate is in column 8. Proofs of our colorings can be found at https://sites.google.com/site/olawaletitiloye/graphcoloring/qacol

An exact simulation of quantum computation with path integral Monte Carlo requires that the number of replicas 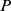 approach infinity [3]. In practice, 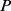 has to be set to a low value. A value of 30 has been used for the traveling salesman problem [8], while we have used values such as 10 and 20 for large instances of the graph coloring problem [9], [10]. Each of the 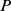 replicas was independently initialized by assigning to each vertex one of the *k* available colors uniformly at random. To alleviate the drawback of using only a small number of replicas, we implemented a similarity control mechanism of preventing directly connected replicas from prematurely becoming too similar, by randomly perturbing a fraction of the spins at critical moments [10]. Specifically, two replicas were considered too similar when one could be made identical to the other by changing the colors of less than 10% of all vertices in one of them [10]. Also, in order to perturb a configuration, we randomly selected 10% of all vertices and assigned a new randomly selected color for each of them [10]. The procedure usually activated itself towards the end of a simulation involving difficult instances. For many of the easier instances, a solution was often already reached before such measures were needed.

**Figure 4:**
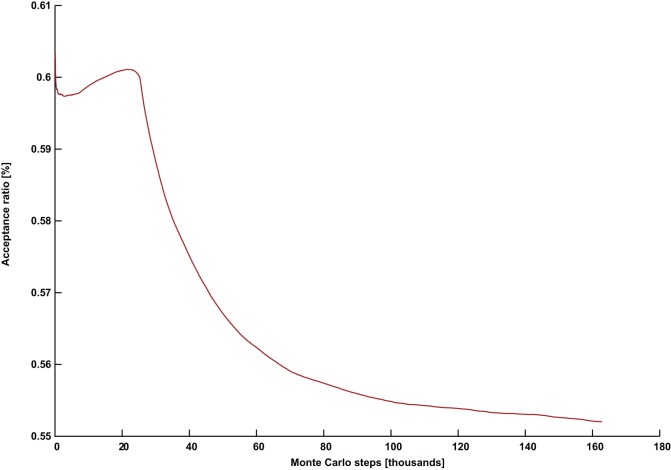
An ineffective simulation for (*G* =  C2000.5, *k* = 146) with an increased temperature *PT*  = 0.35. The acceptance ratio persistently declines in a manner very similar to thermal annealing.

**Figure 5:**
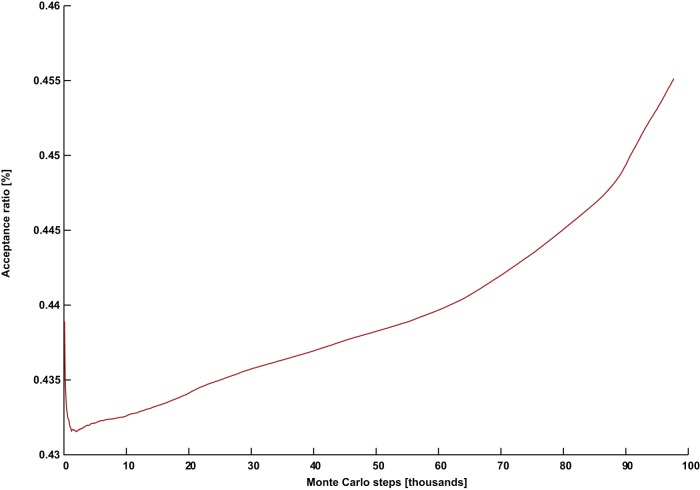
Acceptance ratio plot for (*G* = C2000.5, *k* = 146) with *Γ* = 0.65 and *PT*  = 0.32. A successful simulation shows a continuously rising pattern for the acceptance ratio over time.

**Figure 6:**
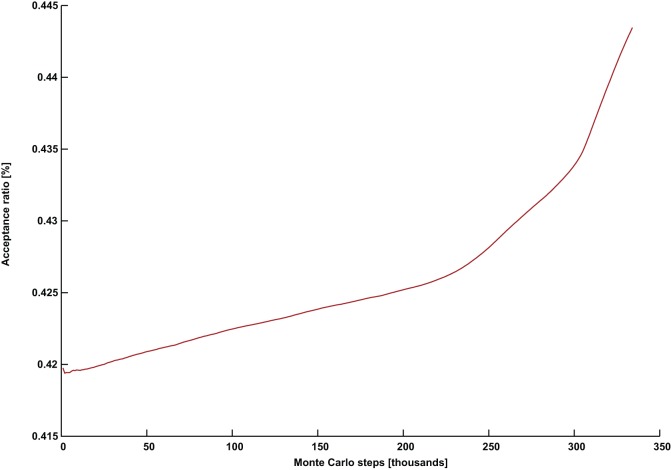
Acceptance ratio plot for (*G* = C2000.5, *k* = 145) with *Γ* = 0.69 and *PT* = 0.32. A successful simulation shows a continuously rising pattern for the acceptance ratio over time.

Our computer hardware setup consisted of 12 CPU cores distributed across two identical desktop computers (having 6 CPU cores each), with each of the 10 replicas running on its own core, and one core left free on each desktop. Each core had a frequency of 2.6GHz, and each desktop had 6GB of RAM. The programming environment was GNU C++ on Linux. Communication between the two computers was achieved with MPI (Message Passing Interface), while intra-computer parallelism was performed with OpenMP (Open Multi-Processing). We considered one Monte Carlo step to be complete after all 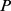 replicas had each completed 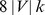 color change attempts [10]. After each Monte Carlo step, each replica received the configuration of directly connected replicas. The configuration of each replica was stored and transmitted as an assignment of vertices to colors. From this, the value of any spin 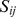 can be deduced. Rather than try to keep track of the instantaneous state of other replicas, each replica simply used an old copy in its computations, until it received an update [10].

**Figure 7:**
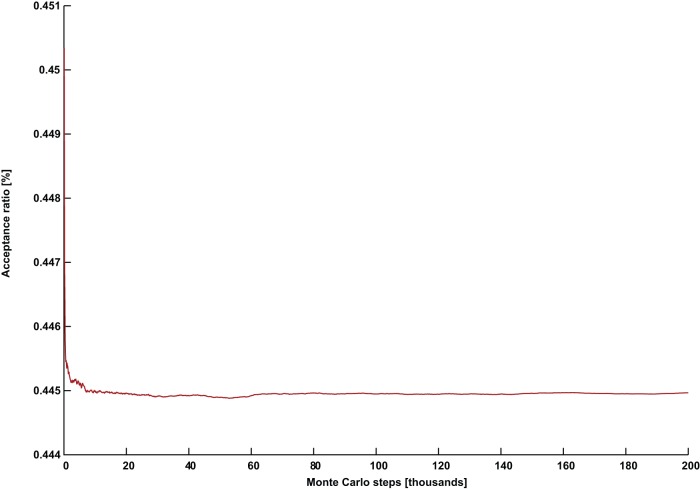
Acceptance ratio plot for (*G* = C2000.5, *k* = 146) with *Γ* = 0.8 and *PT* = 0.32. When the field strength is set higher than the critical value of *Γ* = 0.65, the simulation becomes ineffective. It also shows a persistent stagnation in the evolution of the acceptance ratio.

**Figure 8:**
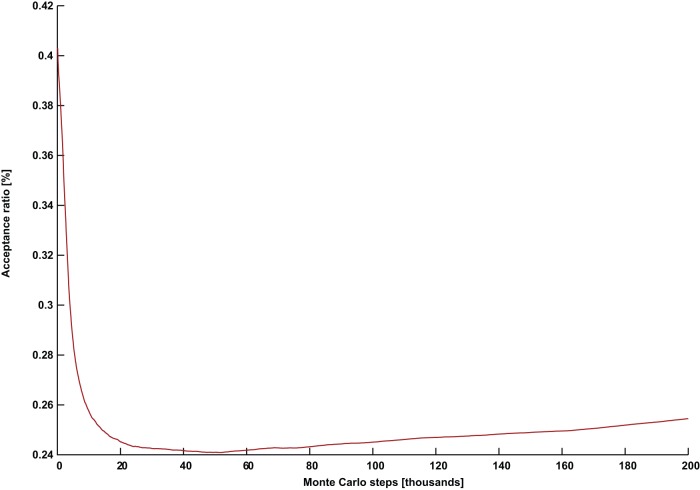
Acceptance ratio plot for (*G* = C2000.5, *k* = 146) with *Γ* = 0.5 and *PT* = 0.32. The simulation becomes ineffective when the field strength is set lower than the critical value of *Γ* = 0.65. The evolution of the acceptance ratio shows a long period of decline and a very weak growth afterwards.

## Results and Discussion

We considered the Erdös-Rényi 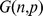, which is a standard model of random graphs in which an *n*-vertex graph is formed by independently including each possible edge with a probability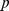. The graph coloring problem is still hard, even when restricted to such random graphs, provided instances are chosen near the uncolorable transition [13], [14], where 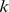 is very close or equal to the chromatic number 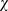. The chromatic number is the smallest possible value of 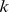 for which a proper coloring exists. In fact, no known polynomial time algorithm is guaranteed to color random graphs with 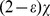 where 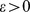 is fixed [15]. We used DIMACS [16] as our source of benchmark graphs, as numerous algorithms have been tested against them. DIMACS random graphs have 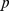 ranging between 0.1 and 0.9, and can all be considered dense in comparison to very sparse graphs that might be 3-colorable [14]. The graph DSJC1000.5 is a member of the 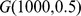
family of graphs. An upper bound of 83 was reported for the chromatic number of DSJC1000.5 in the year 1999 [17], and no algorithm has been able to do better since then. We used our Monte Carlo quantum annealing algorithm given in ref. [10] to easily find colorings for instances in the range 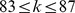, for which the effective temperature 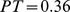 appears to be optimal [9], [10]. Although we set the value of 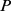 to 10 for all experiments in this paper, different values of 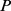 can be used by adjusting 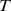 to maintain the same value for 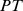 [8], [10]. The acceptance ratio is the number of completed moves (or color changes) divided by the number of attempted moves. We know that 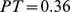 corresponds to a low temperature because the starting acceptance ratio [9], [18] during the Monte Carlo simulation is about 1.3% with 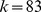. In contrast, a value of 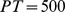 is needed to bring the acceptance ratio to about 50%, which was the starting ratio for comparable experiments with thermal annealing for graph coloring in ref. [18]. Each time we solved for a smaller value of 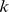, we chose a larger, fixed value for the magnetic field strength 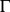 from the range between 0.55 and 0.68, to allow for the increase in difficulty [9].

Since probabilistic counting arguments [18], [19] suggest that colorings with 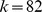 might exist for the 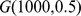 family of graphs, we tried to find the first ever 82-coloring for DSJC1000.5 by setting an increased value for 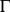. Parameter settings of 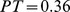 and 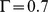 repeatedly failed to produce an 82-coloring, and setting an even larger 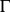 did not improve the situation. When *k* is close to the chromatic number, we are likely to encounter phase transitions in the structure of the solution space [13], [15] characterized by the clustering of solutions, and the subsequent emergence of frozen vertices [14], which might require a change in approach. A further series of experiments with a reduced effective temperature of 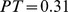 and a field strength 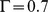 surprisingly produced 82-colorings for DSJC1000.5 with a 100% success rate from 10 independent runs. The successful tuned values of 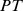 and 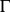 for the DSJC1000.5 graph with 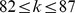 are presented in Fig. 1.

For each successful run with the instance (*G* = DSJC1000.5, *k* = 82), quantum annealing made an average of 5.6×10^11^ color change attempts and 4.4×10^9^ color changes before a solution was found. Our algorithm took an average wall-clock time of 1.2 hours to find an 82-coloring for DSJC1000.5. This is comparable to the computational resources other researchers have dedicated to this problem. For example, in some experiments in ref. [20] and ref. [21], their single-threaded algorithms took up to 10 and 12 hours respectively for each run on the same DSJC1000.5 graph, without reporting any 82 colorings.

Fig. 2 and Fig. 3 show the evolution of the acceptance ratio with time measured in Monte Carlo steps, for the simulations involving (*G* = DSJC1000.5, *k* = 82), with 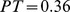 and 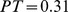 respectively. In the ineffective simulation with the higher temperature in Fig. 2, there is a persistent decline and stagnation of the acceptance ratio. This is also what often happens in a thermal annealing algorithm [18]. However, the successful simulation for the instance (*G* = DSJC1000.5, *k* = 82) with the lower temperature of 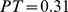 shows an unusual pattern of a continuously rising acceptance ratio (in Fig. 3), which persists until a solution is found. While quantum annealing with a persistently declining acceptance ratio can solve the easier cases of 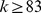 with 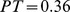 [9], the higher temperature turns out to be problematic for 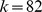, no matter the value of 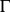. The lower temperature of 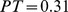 also works for 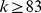, but the required computational effort is unnecessarily increased. Almost our entire past quantum annealing results for random graph coloring [10] used parameter settings which led to stagnating and/or declining acceptance rates. Tuning quantum annealing that way was competitive in its own right. However, parameters that induce a continuously increasing acceptance seem to be necessary for an improved result.

In order to verify that the continuously rising acceptance ratio was not simply due to the perturbation from the similarity control, we first noted that a typical successful run for the simpler instance (*G* = DSJC1000.5, *k* = 83) with 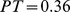 and 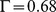 results in about 4000 perturbations out of a total of about 5000 Monte Carlo steps required to solve the problem. A perturbation acts on one replica, while each Monte Carlo step refers to the iteration over P replicas. A declining pattern for the acceptance ratio similar to Fig.2 was observed. However, we found that by setting the reduced temperature of 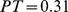 and keeping everything else the same in the aforementioned problem, no perturbations were needed at all to solve the problem, although a much larger number of Monte Carlo steps of about 30,000 were necessary. More importantly, in this case, the acceptance ratio continuously rises as in Fig. 3. Furthermore, even though the more difficult instance (*G* = DSJC1000.5, *k* = 82) with settings 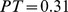 and 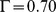 typically requires about 10,000 perturbations out of a total of about 10^5^ Monte Carlo steps, we observed that another successful experiment with the settings of a lower temperature 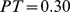, and 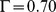 resulted in only 5 perturbations out of about 2.5×10^5^ Monte Carlo steps. Nevertheless, this also produced a continuously rising pattern in the acceptance ratio similar to that in Fig. 3. This shows that the longer simulations with slightly lower temperatures and much less perturbations can still produce a continuously rising acceptance ratio. Therefore we can be confident that the phenomenon is not due to the perturbations used to control similarity.

The continuously rising pattern of Fig. 3 can be induced for other random graphs to produce results that we have not been able to achieve with parameters that exhibit a declining or stagnating pattern. In some cases, our results are the best ever found by any algorithm. For example, in the case of DSJC500.5, a 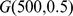 graph that had the upper bound on its chromatic number improved to 48 in the year 1996 [22], we have been able to find 47-colorings for the first time. We did this by setting a reduced temperature of 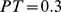 for 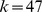, instead of 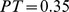 used for 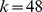 [9], while maintaining the field strength at 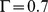. With an appropriately low temperature, and a carefully tuned field strength, quantum fluctuations dominate thermal ones, and quantum annealing is able to escape the deceptive local minima that have confounded all previous algorithms on the (*G* =  DSJC1000.5, *k* = 82) and (*G* = DSJC500.5, *k* = 47) instances for almost two decades. The main competitors of quantum annealing for the coloring of dense graphs are evolutionary algorithms incorporating Tabu local search [17], [21], [23], [24]. Simpler approaches such as thermal annealing [18] and plain Tabu search [25] are generally less competitive for coloring dense random graphs. The preprocessing technique of independent set extraction [19] has been improved [26], [27] to produce good results for very large random graphs, but quantum annealing can also incorporate this idea when necessary.

We have improved on the recent result of 409-colorings in ref. [26] for C2000.9, a 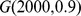 graph, by finding 400-colorings. Quantum annealing also found 145-colorings for C2000.5, a 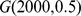
graph, thereby improving on the 146-colorings of ref. [26]. Our results were obtained by selecting parameters that exhibited a continuously rising acceptance ratio. Simulations with parameters that produced a declining acceptance ratio were repeatedly unsuccessful in several independent runs. The graphs C2000.9 and C2000.5 are large, and consist of about 1.8 million and 1 million edges respectively. But unlike in ref. [26], our experiments with C2000.9 and C2000.5 did not need to employ pre-processing by set extraction in order to obtain or improve on the best known results for these particular graphs.

The largest Erdös-Rényi graph from the DIMACS benchmarks is C4000.5, which is a 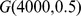
graph with about 4 million edges. It is not often used in experiments due to its very large size. The best result obtained without independent set extraction was a 271-coloring by an evolutionary algorithm incorporating Tabu search [21]. This was recently improved to 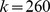 by coloring a residual graph of about 800 vertices obtained after extracting several large independent sets [26]. Encouraged by the superior results of quantum annealing on 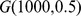, we extracted 163 large independent sets from C4000.5 that were obtained in the experiments of ref. [26] in about 5 days of single processor time. We found 96-colorings of the resulting residual graph of about 1200 vertices by quantum annealing in 12 hours on our hardware setup, thereby providing 259-colorings for C4000.5. Without the aid of set extraction pre-processing, quantum annealing located a 270-coloring in 11 days and a 262-coloring in 4 months. C4000.5 is the only random graph in the DIMACS benchmark for which we had to employ set extraction pre-processing in order to obtain the best results.

Although the graph flat1000_76_0 from the DIMACS benchmarks is not an Erdös-Rényi graph, we have previously observed that quantum annealing required similar parameters to a 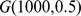 graph when solving instances in the range 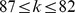 [9]. Specifically, 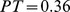 and 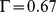 are good parameters for finding 82-colorings of flat1000_76_0, as well as 83-colorings for DSJC1000.5 [9], [10]. Both graphs are also of similar density, and consist of the same number of vertices. Even though flat1000_76_0 is a flat graph with a hidden 76-coloring [28], it tends to behave similarly to DSJC1000.5 when 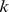 is large enough. For example, algorithms in refs. [17], [20]–[24] produce a similar upper bound for the chromatic number 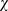 on both flat1000_76_0 and DSJC1000.5, often by using similar parameters and a comparable computational effort for both graphs. It was therefore natural to investigate whether the parameters 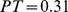 and 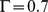 for (*G* = DSJC1000.5, *k* = 82), could also be used to improve the results of the flat graph by solving (*G* =  flat1000_76_0, *k* = 81). Our experiments show that this is indeed the case. After completing our computations, we learned that successful solutions to (*G* = C2000.5, *k* = 145), (*G* = C4000.5, *k* = 259), (*G* = flat1000_76_0, *k* = 81) and (*G* = C2000.9, *k* = 408) have recently been mentioned in ref. [27]. They were obtained by improving the synergy between the set extraction preprocessing in ref. [26] and their main graph coloring algorithm in [24]. Quantum annealing still maintains a lead of eight colors on the very dense C2000.9 by finding 400-colorings. Additionally, our approach is still the only one that successfully solves (*G* =  DSJC1000.5, *k* = 82) and (*G* = DSJC500.5, *k* = 47). The results of our colorings are summarized in Table 1. Additional details such as the parameter settings, number of attempted color changes, wall clock time taken, and the frequency of success can be found in Table 2.

During the quantum annealing of our artificial spin model, even though the state of an individual spin at a future time is highly unpredictable, the acceptance ratio is an easily tracked property describing the collective activity of the spins. We were unable to find any set of parameters producing a declining and/or stagnating acceptance ratio such as in Fig. 2, that nevertheless solves the problem instance (*G* = DSJC1000.5, *k* = 82). Instead the continuously rising pattern in Fig. 3 appears to be crucial for success with that particular instance. Extensive experiments suggest that the temperature 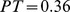 is not low enough for 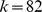, even though it is adequate for the easier instances with 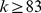, despite the production of a declining and stagnating pattern for the acceptance ratio in those cases. Lowering the temperature to 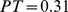 turns out to be useful and important, both in solving the instance (*G* = DSJC1000.5, *k* = 82), and in producing a continuously rising pattern for the acceptance ratio. We also generated five new 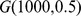 graphs and found that they were all 82-colorable with the same parameter settings of 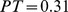 and 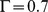. This is not surprising, as Erdös-Rényi graphs with the same values for 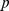
and 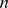 are known to possess very similar characteristics [29].

Additionally, we observed that the larger the graph, the earlier one is forced to start using lower temperatures, even when 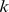 is still far from the chromatic number. For example, the temperature parameter used in ref. [10] to find 147-colorings for C2000.5 was 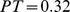, and any attempts to use anything higher causes problems. In our experiments, we used this same temperature value to successfully solve for 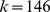 and 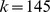, by setting a field strength of 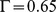 and 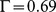 respectively. We also performed several experiments on (*G* = C2000.5, *k* = 146) with a higher temperature 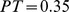, and the field strength maintained at 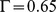. They were all ineffective. Fig. 4 depicts a typical acceptance ratio plot for this experiment. In addition to not locating a solution, the simulations exhibited a persistently declining acceptance ratio reminiscent of thermal annealing. In contrast, the experiments with 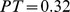 depicted in Fig. 5 and Fig. 6 were effective, and produced a continuously rising acceptance ratio. This strongly suggests that it is necessary for the temperature to be as low as 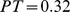. Nevertheless we have observed that random graphs of various sizes with the same probability 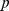 can often be approached with the same (or very close) value for the temperature 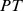. For example, when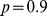, good values for 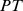 tend to be very close to 0.2 for different values of 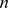, as can be seen from Table 2 and our past graph coloring studies in refs. [9], [10].

The field strength 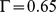 appears to be a special value of the field strength for the instance (*G* =  C2000.5, *k* = 146). Experiments with a higher value of 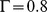 produced an acceptance ratio curve with a persistently stagnating pattern following an initial brief period of decline, as shown in Fig. 7. The simulations with this setting repeatedly failed to reach a solution in several independent runs, even though we allocated three times the amount of Monte Carlo steps as was used for 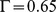. Similarly, simulations with the lower value of 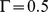 proved to be ineffective. As shown in Fig. 8, the acceptance ratio underwent a long period of decline and only rose very weakly afterwards. The diverging pattern of the acceptance ratio seen in Fig. 5 did not appear. When 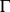 is on either side of 0.65, the behavior of the system starts becoming unfavorable for locating solutions.

The least dense family of 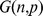 graphs from the DIMACS benchmarks is 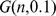. The largest such graph from the benchmarks is DSJC1000.1 with 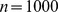. Quantum annealing can match the best algorithms in coloring DSJC1000.1 with 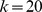: the lowest ever used [9]. However, when we repeated the experiments with various parameter settings, we noticed that unlike denser graphs, we could not find settings which solved the problem with a diverging acceptance ratio. Instead, success for this sparse graph appeared to require a declining acceptance ratio, which started from values as high as 5%. We proceeded to generate several sparser random graphs, some of which were likely to be 3-colorable, and were up to 5000 vertices. A similar behavior to that of DSJC1000.1 was observed during quantum annealing. In fact, the sparser the graph, the closer the performance and behavior of our quantum annealing was to thermal annealing. In our *k*-coloring spin model, all graphs with the same number of vertices possess the same number of spins. But the denser graphs have more constraints, and hence more interaction between their spins. We observed that the appearance of a diverging acceptance ratio correlated with the density of the graph.

By studying the effects of parameter tuning on a Monte Carlo quantum annealing algorithm for the coloring of dense random graphs, we have solved some well known *k*-coloring problem instances that no other approach has been able to. As more insight is gained into parameter tuning, Monte Carlo quantum annealing may be able to improve on other heuristics for different types of combinatorial optimization problems.
